# Recent Advances in Electrochemical Sensors for Caffeine Determination

**DOI:** 10.3390/s22239185

**Published:** 2022-11-25

**Authors:** Žaklina Z. Tasić, Marija B. Petrović Mihajlović, Ana T. Simonović, Milan B. Radovanović, Milan M. Antonijević

**Affiliations:** University of Belgrade, Technical Faculty in Bor, 19210 Bor, Serbia

**Keywords:** caffeine, electrochemical sensor, modifiers, differential pulse voltammetry, real samples

## Abstract

The determination of target analytes at very low concentrations is important for various fields such as the pharmaceutical industry, environmental protection, and the food industry. Caffeine, as a natural alkaloid, is widely consumed in various beverages and medicines. Apart from the beneficial effects for which it is used, caffeine also has negative effects, and for these reasons it is very important to determine its concentration in different mediums. Among numerous analytical techniques, electrochemical methods with appropriate sensors occupy a special place since they are efficient, fast, and entail relatively easy preparation and measurements. Electrochemical sensors based on carbon materials are very common in this type of research because they are cost-effective, have a wide potential range, and possess relative electrochemical inertness and electrocatalytic activity in various redox reactions. Additionally, these types of sensors could be modified to improve their analytical performances. The data available in the literature on the development and modification of electrochemical sensors for the determination of caffeine are summarized and discussed in this review.

## 1. Introduction

Caffeine (1,3,7-trimethylxanthine) belongs to the group of natural purines. Among humans, caffeine has a long tradition of consumption. It is a natural alkaloid found in numerous plant species such as coffee beans, tea leaves, cocoa beans, and guarana berries. Additionally, caffeine (CF) is an ingredient of energy drinks as well as pharmaceutical drugs. Since caffeine has positive effects on physical endurance, improves concentration, reduces oxidative stress, and relieves fatigue and headache, it is used in significantly large quantities [1,2]. However, the excessive use of caffeine leads to adverse effects such as anxiety, increased blood pressure, and cardiovascular diseases [3]. As a result of the widespread consumption of caffeine-containing products, through urine, caffeine enters municipal wastewater [4]. About 20 metabolites are known that arise from the biotransformation of caffeine in the liver. The primary ones are dimethylxanthines (paraxanthine, theobromine, and theophylline), followed by dimethyl- and monomethyluric acid as well as uracil derivatives, while about 1–5% of unchanged caffeine is excreted through urine [5]. Due to the improper disposal of medicines and other waste, caffeine can reach underground and surface waters [6]. Therefore, the detection of caffeine in very low concentrations in different media is very important.

A large number of analytical techniques are suitable for caffeine detection, including chromatographic, spectroscopic techniques [7,8,9], and electrochemical methods [10]. Electrochemical techniques are characterized by numerous advantages over other methods such as their simplicity of the instrumentation, high sensitivity, and the short time needed for the analysis. Instruments can also be mobile, which is important for environmental monitoring [11]. Along with an adequate method, it is necessary to develop a suitable electrochemical sensor. Electrodes made of precious metals, such as platinum and gold, enable the achievement of very favorable kinetics of electron transfer and have a wide potential window in the positive areas of potential. The low overvoltage of hydrogen evolution of these electrodes limits their use in the negative potential region (the cathodic limit is in the interval from −0.2 V to −0.5 V depending on the pH). Moreover, other substantial problems are posed by high background currents, which are a consequence of the formation of oxides on the surface of the electrode or the adsorption of hydrogen. In addition to classic carbon materials, graphite, and glassy carbon, newer carbon materials, such as fullerenes, carbon fibers, carbon nanotubes, and boron-doped diamond, are used for the production of working electrodes. Various properties of carbon as an electrode material—including its chemical stability, significant active surface, and the possibility of modifying its surface—set it apart from the others. Additionally, the greatest advantages of carbon electrodes are its price, wide potential window, relative electrochemical inertness, and electrocatalytic activity in different oxidation–reduction reactions [12]. Concerning the use of low-cost carbon materials as potential sensors, in the review paper published by Tasić et al. [13], the application of a pencil graphite electrode as a sensor for the determination of pharamceutically important components is described. The same group of authors [14] prepared an electrochemical sensor using recycled carbon from spent batteries, which successfully detected L-tryptophan in milk and apple juice samples. In addition, Liu et al. [15] prepared a modified glassy carbon sensor using porous carbon and polyaniline for the determination of insecticides. In order to obtain porous carbon, the researchers used raffia biomass. Researchers are continuously engaged in the challenges of using environmentally friendly materials and synthetic processes when developing sensors.

The importance of carbon electrodes is reflected in their potential to be modified in a simple way. Some of the main goals for which the electrodes are modified [16] are the reduction of the limit of detection (LOD) by accumulating analytes on the surface of an electrode, the enhancement of electrochemical signals by the application of an electrocatalyst, moving and separating overlapping signals, the improvement of the kinetics of electron transfer, and the improvement of general analytical conditions. The modification of the electrodes’ surfaces can be performed using various procedures, e.g., chemisorption, the formation of covalent bonds between specific functional groups on the surface of the electrode and the reagent, by coating the electrode with a polymer film (including the electropolymerization of monomers). The most frequently applied modifiers are metals, metal oxides, metal complexes, surfactants, and, nowadays, nanomaterials. Oxides and metal complexes act as electrocatalysts due to the possibility of the change of their valence state; surfactants improve the surface characteristics of the electrode, while nanomaterials act as catalysts and increase the active surface of the electrode [17]. On the other hand, researchers encounter problems because the utilization of certain nanomaterials as modifiers requires the use of toxic reagents, and the synthesis process is time consuming. In addition, the modification of electrochemical sensors with metal–organic frameworks (MOFs) is very important in this field. Their properties, such as high porosity and large specific surface area, lead to the improvement of the electrochemical signal of the target analyte. According to Yin et al. [18] and Ding et al. [19], these types of sensors were very successful in the determination of antibiotics and glucose, respectively. In addition, Minh et al. [20] and Varsha et al. [21] described the preparation of novel MOF-modified glassy carbon electrodes for the determination of caffeine.

**Figure 1:**
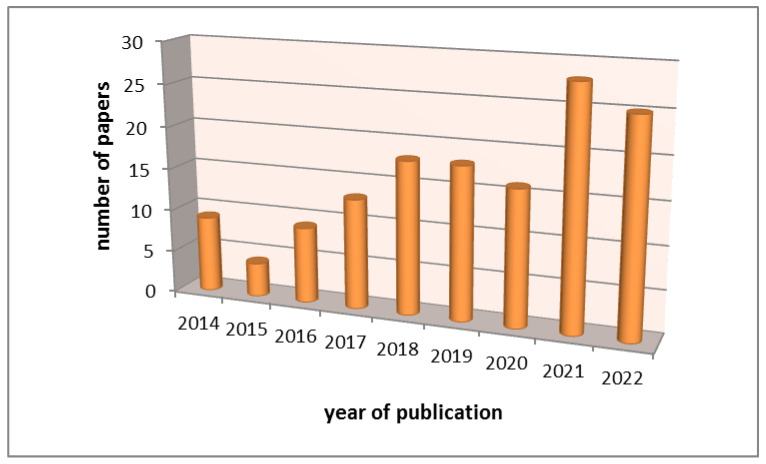
The number of published papers dealing with electrochemical sensors for the determination of caffeine from 2014 to 2022 based on Scopus database.

An introspection of the Scopus database reveals a growing trend in the number of published papers dealing with electrochemical sensors for the determination of caffeine (Figure 1). In addition to the research papers, two book chapters were published as well. Petrucci et al. [22] presented the development of sensors based on graphene oxide and reduced graphene oxide for the determination of methylxanthines. Besides methylxanthines, porous reduced graphene oxide was a good modifier for a glassy carbon electrode for the determination of gliadin in food samples [23]. The significance and importance of graphene as a material for the preparation of sensors is highlighted in the paper by Murthy et al. [24]. Curulli [25] summarized the data on the use of nanomaterials including carbon-based materials as sensors for food analysis. In addition, Mohamed [26] discussed screen-printed carbon electrodes and their applicability in pharmaceutical analysis. Considering that caffeine and other methylxanthines are also found in medicines, it is important to show the application of sensors in that area, too. The aim of this review is to highlight the progress in the development of electrochemical sensors from the year 2014 to the present day and to provide an adequate tool to researchers working on improving the characteristics of electrochemical sensors for the determination of caffeine in various matrices. This time frame is chosen in order to present the most recent data and avoid repeating the data presented in the review published by Svorc [10].

## 2. Discussion

### 2.1. Electrochemical Sensors Based on Carbon Materials for Caffeine Determination

#### 2.1.1. Electrochemical Sensors Based on Glassy Carbon Electrode

The importance of unmodified and modified carbon electrodes is reflected in their application for the determination of heavy metal ions [27] and organic compounds including pesticides, dyes, and pharmaceutical active substances [26]. Researchers have investigated the effect of experimental conditions including the solution pH, scan rate, and the concentration of target analytes on the performance of modified sensors. In general, the current response of the CF was higher and the peak shape was superior in the modified compared to the bare electrochemical sensors.

Okutan et al. [28] developed a new way of modifying glassy carbon electrodes (GCE) with graphene-based zirconia nanocomposite. This modification improved the electrode’s characteristics as a sensor for caffeine detection. Among different acidic media, 0.1 M of sulfuric acid provided the best results. The correlation between the scan rate change and subsequent peak current change indicated that caffeine oxidation is a diffusion-controlled process. The correlation between the peak potential and scan rate indicated that the electron transfer number of 3.666 was approximately the same as the number of electrons that participate in the caffeine oxidation reaction, which is 4. Citric acid, NaCl, glucose, fructose, and sucrose, compounds often found in beverages, were studied as the interferences and the electrode showed good sensitivity. After 15 days in a refrigerator, 74.4% of the current response remained.

Amalraj et al. [29] developed an electrochemical sensor for caffeine detection based on ZnCo_2_O_4_ modified with Pt nanoparticles. It was useful for detection in beverage samples. The structure of this electrode consisted of Pt nanoparticles that were deposited on self-assembled nanosheets that formed a 3-D flowerlike morphology. This complex structure was deposited on a glassy carbon electrode, so the electrode used for sensing could be characterized as Pt@ZnCo_2_O_4_/GCE. The optimal amount of Pt@ZnCo_2_O_4_ was found to be 6 µL and several electrolytes were tested but sulfuric acid was chosen as the most suitable one. Cyclic voltammetry (CV) results showed an improved response of the modified electrode in comparison with bare GCE. The increase in the response followed the order GCE 1.56 V 13.06 µA, ZnCo_2_O_4_/GCE 1.53 V 15.14 µA, Pt/GCE 1.52 V 16.38 µA, and Pt@ZnCo_2_O_4_/GCE 1.49 V 20.77 µA. There was a linear correlation between the peak current and scan rate, so it was concluded that the electrooxidation process was surface-controlled. Numerous interfering substances—such as glucose (GLU), glycine (GLY), Zn^2+^ ions, ascorbic acid (AA), Cu^2+^ ions, citric acid (CA), K^+^ ions, Na^+^ ions, hypoxanthine (HYX), amino acetic acid (AAA), glutamic acid (GTA), sucrose (SUC), and fructose (FRU)—were used, and the electrode was still efficient in the selective detection of CF. The electrode also showed good stability, repeatability, and reproducibility.

Saravanakumar et al. [30] tested the possibility of using a modified GCE electrode. Copper-4,4′-bipyridine-trimesic acid-interlinked MOF (CBT-MOF)-derived carbon composites obtained by carbonization of the pristine CBT-MOF at different temperatures of 500 °C (CBT-500), 600 °C (CBT-600), and 700 °C (CBT-700) under inert conditions, were decorated on glassy carbon electrodes. Carbonization at 500 °C provided the electrode with the best characteristics, in which the surface activity was substantially increased, and the CV peak had the lowest potential value and high current density. The superiority of the CBT-500, with higher conductivity and active surface of the electrode, was attributed to the existence of Cu(I), whereas in the other cases both Cu(I) and Cu(II) were detected. The effect of the scan rate change indicated that the electrochemical process was under diffusion control and pH change was also observed to be an important parameter, whereas at pH 7, the maximum peak current was detected. Selectivity was confirmed in the presence of citric acid, ascorbic acid, sucrose, and glucose, as well as the inorganic ions Zn^2+^, Mg^2+^, and Cu^2+^. After 7 days, a stability study showed a 95% amperometric current response.

Singh et al. [31] used gadolinium molybdate Gd_2_(MoO_4_)_3_ (Gam) that was synthesized by a hydrothermal method for the modification of a glassy carbon electrode to detect caffeine and theophylline. According to the linear sweep voltammetry (LSV) and electrochemical impedance spectroscopy (EIS) data, the treatment temperature of 150 °C provided the best results, lower resistance, and a more effective charge transport between the electrode and electrolyte; thus, it was used for further experiments. The oxidation current detected in the CV measurements depended on the amount of the modification agents, so 5 μL of 1 mg mL^−1^ Gam-modified GCE was chosen. It was also essential to determine the optimal pH value, which turned out to be pH 7 due to the lower peak potential value and higher peak current value as could be concluded form the differential pulse voltammetry (DPV) results. The oxidation peak potentials in the CV curves for caffeine and theophylline shifted towards the higher potentials with the increase in the scan rate, which suggested simultaneous diffusion at the electrode’s surface. The parameters obtained by the application of DPV for caffeine detection under the determined optimal conditions are presented in Table 1. After DPV, the amperometric i-t technique was applied and again the linear correlation between the current detected and concentration of caffeine or theophylline was observed. The potential chosen for caffeine detection was 1.38 V and 1.03 V for theophylline. So, this electrode could be used for caffeine and theophylline detection using the amperometric technique due to its high sensitivity and fast response. This electrode successfully detected caffeine in green tea leaves and coca with and without spiking and in theophylline after spiking.

Sebokolodi et al. [32] modified a glassy carbon electrode with activated carbon nanofibers (CNF) in order to improve its characteristics as a caffeine sensor. The activation of the CNFs was achieved by acids, namely, HNO_3_ and H_2_SO_4_. They applied the drop-coating method and used several methods—such as X-ray diffraction spectroscopy, Raman spectroscopy, Fourier transmission infrared spectroscopy (FTIR) and electron microscopy, field emission scanning microscopy (FESEM), and transmission electron microscopy (TEM)—to investigate the differences in the behavior and morphology of pristine and acid-activated CNFs. It was concluded that acid activation led to the formation of a greater number of carboxylic acid sites that improved functionalization and dispersibility; however, the structure was destroyed or distorted. CV, EIS, and square wave voltammetry (SWV) measurements showed that the modified electrode possessed improved electrocatalytic characteristics. SWV proved that the oxidation of caffeine occurred at lower potential, which was likely due to an increase in the electroactive surface area, and the current response was about two times higher. The determined values of the peak current and potential were 17 µA at 1.44 V for bare GCE and 40 µA at 1.35 V for CNF-GCE. Acetaminophen (AC), glucose, and acetylsalicylic acid (AS) were used as interferences, and it was noticed that only glucose—due to complex formation—led to a decrease in peak current. Thus, this sensor could be used for the detection of caffeine in pain-relief medicines.

Concerning the enhancement of the properties of GCE for the simultaneous determination of caffeine, vanillin (VAN), and 5-O-caffeoylquinic acid (5-CKA), Yigit et al. [33] used graphene as a modifier. In addition, the authors compared the performance of modified and bare GCE for caffeine oxidation. According to the cyclic voltammetry curves, the CF peak intensity was higher (11.22 µA) on the GR/GCE than (2.12 µA) on the GCE. The developed sensor combined with optimized square wave-adsorptive-stripping voltammetry (SWAdSV) was successful in detecting the three compounds in 0.1M PBS (pH 2.5) individually as well as simultaneously. The mechanism of caffeine oxidation on GR/GCE was consistent with similar observations in the literature, involving four electrons and four protons [10]. The prepared sensor was tested after storage for 21 days and the peak intensities of 5-CKA, VAN, and CF were 93.52, 95.44, and 96.23% of their initial values. All results showed that it was a stable and sensitive sensor that could be successfully applied in real food and beverage samples.

Jiang et al. [34] modified GCE by electrodeposition of nitrogen-doped graphene (NGR) and nitrogen-doped carbon nanotubes (NCNTs) (ENGR–NCNTs/GCE), while Fernandes et al. [35] prepared MnFe_2_O_4_@N-CNT/GCE using nitrogen-doped carbon nanotubes (N-CNT) functionalized with MnFe_2_O_4_ nanoparticles (MnFe_2_O_4_@N-CNT/GCE). The developed ENGR–NCNTs/GCE were tested for the simultaneous determination of caffeine and vanillin in an acidic (0.01 M H_2_SO_4_) solution [34]. Fernandes et al. [35] compared the analytical performance of bare GCE, N-CNTs/GCE, and MnFe_2_O_4_@N-CNT/GCE for the detection of CF, AA, and AC in a H_2_SO_4_/Na_2_SO_4_ buffer solution. According to the results obtained by cyclic voltammetry, the authors indicated that MnFe_2_O_4_@N-CNT/GCE significantly improved the intensity of the CF current peak by about 52% compared to bare GCE. It was assumed that the used nanoparticles behaved as a catalyst towards the oxidation of CF, as well as towards ascorbic acid and acetaminophen. In addition, the modified electrodes were tested for the simultaneous determination of these three analytes. MnFe_2_O_4_@N-CNT/GCE was distinguished as a suitable sensor for the simultaneous research of CF, AC, and AA since three peaks were clearly separated, in contrast to bare GCE, in which the peaks overlap. More details about the research are presented in Table 1. In accordance with the data in these papers, it can be surmised that doping carbon materials with nitrogen and nanoparticles contributes to better electrocatalytic activity and a larger specific surface area compared to bare GCE. The electropolymerization of luteolin and functionalized multi-walled carbon nanotubes on GCE (Lt/fMWCNT/MGCE) was the subject of the work performed by Amiri-Aref et al. [36]. Four compounds, including caffeine, xanthine, noradrenaline, and acetaminophen, were quantified in phosphate-buffered solution by this sensor. For the determination of both caffeine and theophylline (TP), Wang et al. [37] also modified a glassy carbon sensor by electropolymerization using nitrogen-doped carbon nanotubes (N-CNTs) decorated with poly(L-cysteine) (PLCY). The analysis of the surface of the PLCY/N-CNT/GCE by a scanning electron microscope (SEM) and TEM indicated the porous structure of the formed polymer film with a large surface area and numerous active sites for the CF and TP oxidation. Cyclic voltammetry, differential pulse voltammetry, and chronocoulometry (CC) were chosen for examining the electrochemical behavior of methylxanthines. A remarkably better shape peak was observed on the modified electrode than on bare GCE. The current peaks of CF and TP depended linearly on the square of the scan rate, indicating a diffusion-controlled process in 0.01M H_2_SO_4_-Na_2_SO_4_. The LOD values are shown in Table 1. The applicability of the prepared sensor for the determination of CF and TP in real beverage samples was demonstrated. In addition, GCE modified by electropolymerization with 4-amino-5-hydroxy-2,7-naphthalenedisulfonic acid showed a sensory capability for the determination of theophylline in phosphate buffer solution (pH 7) and in human blood plasma and urine samples [38]. Wu and his coworkers [39] suggested that Langmuir–Blodgett film (LB) could be used to fabricate the homogenous and controlled films of Nafion-nitrogen doped carbon nanotubes (Nafion-NCNTs) for the determination of caffeine. The conducted measurements showed that the LB films of Nafion-NCNT-modified GCE can significantly improve the electrochemical response to CF and that the modified surface has good stability. Caffeine was detected from commercial tea (soaked in distillated water (90 °C)), which was filtered and directly added to the supporting electrolyte (0.1 M H_2_SO_4_ (pH = 0.94)). Cyclic voltammetric measurements confirmed that the positively charged caffeine accumulated on the surface of the electrode modified with homogeneous LB films of Nafion. However, the best result was obtained for the Nafion-NCNTs-LB/GCE, with the maximum current response of 12.87 μA for caffeine at 1.42 V. This behavior can be explained by considering that the addition of NCNTs leads to the increased surface area of the electrode and its enhanced conductivity. The oxidation peak current response became greater as the number of modified layers increased. The same trend was observed when increasing the scan rate; hence, it was concluded that the electrode reaction of caffeine was a mixture of diffusion- and adsorption-controlled processes.

Sundramoorthy et al. [40] modified GCE with a nanocomposite consisting of polymerized 8-aminopyrene-1,3,6-trisulfonic acid (APTA) and single-walled carbon nanotubes (SWCNTs). The results obtained using the poly(APTA)/SWCNT-modified GCE are presented in Table 1. This electrode proved to be very stable and reliable because the peak current changed only 6% after 100 cycles and the standard deviation between 5 different sensors prepared in the same way was only 2.4%. Dopamine (DA) and ascorbic acid did not interfere with CF detection. Minh et al. [20] used the microwave (MW) method to synthesize MOF-199, which was further utilized as a modifier for GCE (MW-MOF-199/Naf-GCE). The sensitivity characteristics of the thusly prepared electrode were investigated for the determination of CF and paracetamol. Due to the presence of aromatic rings, carboxyl groups, and Cu(II) in MOF-199, it was assumed that there was an interaction between these groups and the aromatic rings of the target analytes. All this contributed to a better voltammetric response of CF compared to the bare GCE. Based on the effect of the solution pH and the scan rate on the potential of the oxidation peak, Minh et al. [20] indicated that the same number of protons and electrons participate in the irreversible reaction of CF oxidation. In addition, the proposed sensor using the DPV method was tested for the analysis of pharmaceutical drugs containing CF and paracetamol. The obtained results indicated that MW-MOF-199/Naf-GCE and DPV are suitable for the analysis of such real samples and could be an adequate substitute for expensive methods.

The aim of the work of Tyszczuk-Rotko and Beczkovska [41] was to improve the analytical performance of a glassy carbon electrode as a sensor for the determination of CF in an acidic medium as well as in pharmaceutical formulations and beverages. For this, they modified GCE with a lead film and a Nafion layer. Two electrochemical methods, cyclic voltammetry and differential pulse adsorption-stripping voltammetry, were performed and according to the results, the larger active surface area of modified GCE with Nafion/Pb film provides a significantly improved voltammetric response of the CF. In addition, the authors indicated that the modification process itself affected the characteristics of the sensor. The potential and deposition time as well as the thickness of the lead film were the main parameters. After optimizing these parameters, the authors suggested a 30 s deposition time at a potential of −1.4 V and the lead concentration of 7.5 × 10^−5^ mol L^−1^ for the best results. 

A dispersion of carbon black (CB), graphene oxide (GO), copper nanoparticles (CuNPs), and poly(3,4-ethylenedioxythiophene)-poly(styrenesulfonate) (PEDOT:PSS) was created; dropped onto the GCE surface; and formed GO-CB-PEDOT:PSS/GCE [42]. The developed sensor, GO-CB-PEDOT:PSS/GCE, for the detection of isoproterenol, acetaminophen, folic acid, propranolol, and caffeine was tested by applying square wave voltammetry and cyclic voltammetry in 0.1M PBS (pH 7). Under the investigated conditions, one anodic peak of caffeine was observed on the modified GCE. In addition, the intensity and shape of the current response were much better on the GO-CB-PEDOT:PSS/GCE than on the bare one due to the higher active surface. By using this sensor and optimized SWV, the authors successfully determined five analytes in their mixture without overlapping of the current peaks. The micromolar detection limit value is shown in Table 1. In addition to the established selectivity, the stability and reproducibility of GO-CB-PEDOT:PSS/GCE were good, with RSDs lower than 10%.

**Figure 2:**
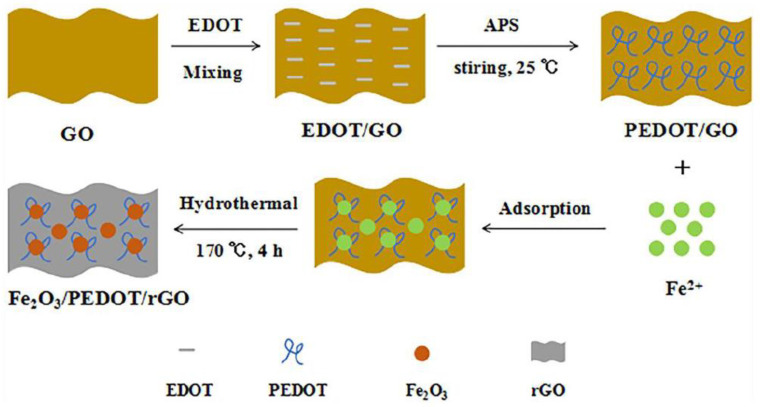
Schematic illustration for the preparation of Fe_2_O_3_/PEDOT/rGO composite. Reprinted from [43].

A nanocomposite containing iron oxide (Fe_2_O_3_), reduced graphene oxide (rGO), and poly(3,4-ethylenedioxythiophene) (PEDOT) was synthesized (Figure 2) and used as a modifier for a glassy carbon electrode [43]. Thus, the prepared Fe_2_O_3_/PEDOT/rGO/GCE was tested for caffeine detection in Britton–Robinson buffer as well as in beverage samples. 

**Figure 3:**
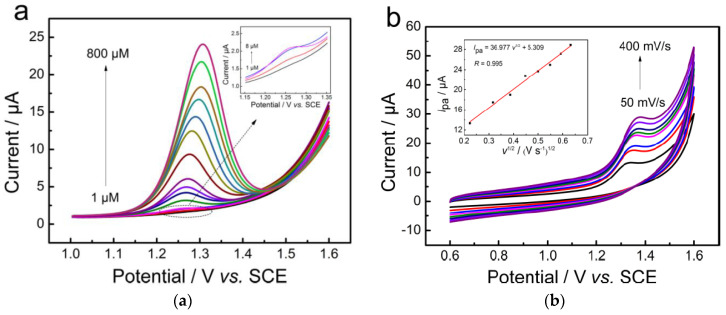
(**a**) DPVs of the Fe_2_O_3_/PEDOT/rGO obtained in the BR buffer solution (pH = 1.8) containing different concentrations of caffeine and (b) CVs of Fe_2_O_3_/PEDOT/rGO/GCE in the presence of 0.1 mM of caffeine in BR buffer solution (pH = 1.8) at different scan rates (50–400 mV s^−1^). Inset: plots of peak current vs. the square root of scan rate (v^1/2^). Reprinted from [43].

Gao et al. [43] compared the sensing performance of bare GCE, Fe_2_O_3_/rGO/GCE, and Fe_2_O_3_/PEDOT/rGO/GCE and observed the best CF peak shape and the highest peak intensity on Fe_2_O_3_/PEDOT/rGO/GCE. Poly(3,4-ethylenedioxythiophene) improved the active surface area and conductivity of the electrode, which contributed to its good performance. According to the electrochemical behavior of CF on Fe_2_O_3_/PEDOT/rGO/GCE, it was an irreversible reaction. The peak intensity of CF increased with the scan rate as well as the concentration (Figure 3). The obtained value of the LOD is presented in Table 1. Following an investigation of its stability, reproducibility (RSD 3.4%), and selectivity, the prepared Fe_2_O_3_/PEDOT/rGO/GCE showed good characteristics. After a storage period of 30 days, the peak intensity remained 90% of the initial value. In the presence of various ions, including K^+^, Na^+^, Mg^2+^, Fe^3+^, Zn^2+^, Cu^2+^, NO_3_^−^, SO_4_^2−^, Cl^−^, and CO_3_^2−^, as well as glucose, D-fructose, caffeic acid, citric acid, ascorbic acid, and L-glutamic acid, the electrode showed good selectivity in the determination of caffeine.

Velmurugan et al. [44] modified GCE with rGO decorated with a core–shell-like structure of Cu_2_O nanocubes enfolded by Co(OH)_2_. Interferences such as ascorbic acid, citric acid, fructose, sucrose, and glucose were introduced, and the electrode showed good selectivity towards caffeine. Good electrode characteristics were maintained after five successive measurements, with an RSD of 3.2%; after 5 weeks, there was a 91.2% initial current response, and five different electrodes showed an RSD of 3.7%.

An electrode based on a glassy carbon electrode modified with an attapulagite/nafion film (AT/Naf/GCE) was developed in order to electrochemically determine caffeine [45]. DPV and CV were the methods for studding the ionic exchange properties and conductivity of AT/Naf/GCE. The investigation showed that caffeine yielded an irreversible oxidation peak when the electrode was modified by a thin film of AT and Naf, which proved that the presence of both on the GCE favorably catalyzed the caffeine’s oxidation. Measurements were conducted to determine the repeatability of the AT/Naf/GCE sensor for a 1 × 10^−5^ M caffeine solution under optimized conditions. A relative standard deviation of 3.14% calculated from four successive measurements indicated the sensor’s good repeatability. The pharmaceutical formulation Brupanax was chosen as a real sample of a commercial product to test the proposed sensor. The oxidation peak of caffeine was proportional to the added caffeine concentration with 92% recovery.

Jagadish et al. [46] used zinc oxide nanoparticles as a modifier for a glassy carbon electrode (ZnONPs/MGCE) to improve the analytical performance of GCE for the determination of CF. A higher voltammetric peak at lower potential value of CF was observed on ZnONPs/MGCE compared to bare GCE due to electrostatic interactions between the negatively charged CF and the positively charged zinc oxide nanoparticles. Additionally, the large surface area and better conductivity improved the current response of CF on the ZnONPs/MGCE. In order to develop a new platform for the detection of CF and TP, a poly(L-aspartic acid)/functionalized multiwalled carbon nanotube composite was prepared and used as a modifier of GCE (P(L-Asp)/f-MWCNTs/GCE) [47]. The application of the composite-modified GCE enabled a higher current peak of the oxidation of the target analytes due to a larger specific surface area and improved electron transfer. Further, for the determination of CF and TP, Shu et al. [48] used a composite film of poly(folic acid) and graphene for the modification of GCE (PFA/GR/GCE). The research groups of Shu et al. [48] and Mekassa et al. [47] demonstrated good analytical performances of their prepared sensors for the simultaneous determination of CF and TP in phosphate buffer solution (pH 4.5). The oxidation peak potentials of both analytes were very similar (Table 1). By investigating the influence of the pH value of the support solution on the peak potentials, both Shu et al. [48] and Mekassa et al. [47] suggested that the same number of protons and electrons were included in the redox mechanism of TP. On the other hand, increasing the pH value did not cause a shift in the peak potential of CF, which indicated that the protons were not involved in the redox process. In addition, GCE was modified using composite films of poly(diallyldimethylammonium chloride), multiwalled carbon nanotubes, and Nafion (Nafion/PDDA-MWCNT/GCE) [49] as well as poly(3,4-ethylenedioxythiophene) (PEDOT), MWCNT, and Nafion [50] for the quantification of caffeine in beverages and pharmaceutical samples. The determination of caffeine in tea samples was successful with the prepared polydopamine–gold nanocomposite on a GCE (PDA/AuNPs/GCE) sensor [51]. The achieved values of the LOD are shown in Table 1. Nafion in combination with graphene was used to improve the GCE properties (GR-NF/GCE) for the determination of caffeine, paracetamol, and aspirin in pharmaceutical samples [52]. The developed sensor with the appropriate method (square-wave adsorptive anodic-stripping voltammetry) was successful in the individual analysis of these three analytes as well as in their mixture. The sensor’s good repeatability (RSD 5.72%) and good stability after 21 days of storage (RSD 6.22%) made GR-NF/GCE a possible alternative to more expensive and demanding analytical methods.

Anastasiadi et al. [53] used an anodically pretreated glassy carbon electrode as a sensor for the determination of caffeine and paraxanthine (PX) in human saliva. According to cyclic voltammetric measurements, the authors indicated that the shape and intensity of the current response depended on the pH value of the supporting electrolyte. Among the used electrolytes (HNO_3_, H_2_SO_4_, H_3_PO_4_, and Britton–Robinson buffer) in different concentration ranges, 0.1M H_2_SO_4_ was selected as the most suitable for this investigation. In order to examine the selectivity of anodically pretreated GCE, theophylline and theobromine (TB) were added to the electrolyte as interferences because they could be found in the saliva. The obtained DPV showed that the oxidation potentials of TB and TP were the same as those of CF and PX, respectively.

A composite film consisting of poly (4–vinylpyridine) (P4VP) and multiwalled carbon nanotubes (MWCNT) was used as a modifier to improve the sensory properties of GCE (P4VP–MWCNT/GCE) [54]. In the research, cyclic voltammetry and differential pulse voltammetry were performed, and the optimized parameters are shown in Table 1. The developed sensor showed the ability to determine CF individually and simultaneously in the presence of aspirin in phosphate buffer solution. The presence of paracetamol as a third analyte did not interfere with the CF and aspirin signals on P4VP–MWCNT/GCE. A glassy carbon electrode was modified with 7-amino-4-hydroxynaphthalene-2-sulfonic acid (poly(7A4HN2SA)/GCE), and was used for the determination of caffeine [55]. The current response of CF on the modified GCE was improved compared to the bare GCE. Additionally, the peak potential of CF was about 40 mV lower on poly(7A4HN2SA)/GCE than on GCE due to the electrocatalytic effect of the formed polymer film towards caffeine oxidation. The fabrication process of poly(7A4HN2SA)/GCE was very simple, which was an advantage of this sensor. The presence of sucrose, fructose, taurine, and glucose did not influence the CF current response, while riboflavin and ascorbic acids had negative and positive effects, respectively. This means that poly(7A4HN2SA)/GCE with DPV can detect caffeine in samples without or with a lower concentration of riboflavin.

Hallaj et al. [56] prepared a new electrochemical sensor for the determination of caffeine in a 0.1 M phosphate buffer solution. The base of the sensor was a glassy carbon electrode whose surface was additionally modified with graphene, SiC nanoparticles, and [Cu(pidc)(apim)]_2_. Its characteristics were also tested for caffeine detection in real samples (Table 1). By comparing the cyclic voltammograms on bare GC, GC/Gr, and GC/Gr/SiC-S@[Cu(pidc)(apim)]_2_ in PBS with the addition of caffeine, the electrooxidation of caffeine on the GC/Gr/SiC-S@[Cu(pidc)(apim)]_2_ electrode took place at a lower potential than other sensors. Accordingly, the authors indicated that the Cu(I)-complex was an electron transfer mediator for caffeine oxidation and proposed the following mechanism:

Cu(I)Cu(I) ⇔ Cu(I)Cu(II) + e(1)

Cu(I)Cu(II) + Caffeine ⇔ Cu(I)Cu(I) + Oxo-Caffeine(2)

Cu(I)Cu(II) ⇔ Cu(II)Cu(II) + e(3)

Cu(II)Cu(II) + Caffeine ⇔ Cu(I)Cu(II) + Oxo-Caffeine(4)

Another modified GCE with nicotinic acid hydrazide anchored on graphene oxide (NAHGO) was fabricated by Jose et al. [57]. Its sensory characteristics in the determination of caffeine were tested using voltammetric techniques. Various techniques such as FT-IR, X-ray powder diffraction (P-XRD), Raman spectroscopy, SEM, X-ray photoelectron spectroscopy (XPS), and thermogravimetric analysis (TGA) were performed to study the morphology of NAHGO. Based on the results, the authors concluded that epoxy, alcohol, carboxylic acid, pyrrole, and pyridine nitrogen groups were the main interaction sites between NAHGO and caffeine. In addition, the higher active surface of NAHGO (0.29 cm^2^) than GCE (0.078 cm^2^) contributed to the better electrochemical response of the CF. 

The electrochemical behavior of Co_3_O_4_/GCE-Nafion as a sensor for the quantification of CF under different conditions was demonstrated [58]. Buffer solutions including acetate, phosphate, and Britton–Robinson were used as supporting solutions in this research. Acetate buffer was selected as optimal, attaining the most pronounced current peak of caffeine. Differently from the previously mentioned research, Kumar et al. [58] used chronoamperometry and square wave voltammetry; their obtained LODs are shown in Table 1.

**Figure 4:**
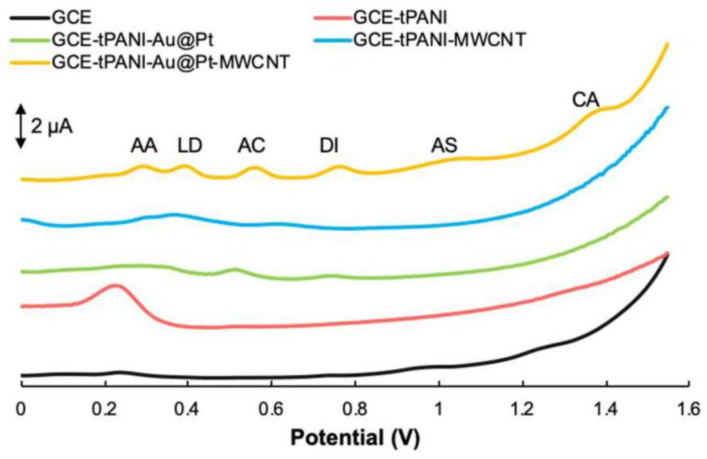
DPVs for the electrochemical oxidation of ascorbic acid (AA) (150 µM), levodopa (LD) (15 µM), acetaminophen (AC) (15 µM), diclofenac (DI) (15 µM), acetylsalicylic acid (AS) (150 µM), and caffeine (CF) (150 µM) at the blank GCE (black line), tPANI-modified GCE (red line), tPANI-Au@Pt-modified GCE (green line), tPANI-MWCNT-modified GCE (blue line), and tPANI-Au@Pt-MWCNT-modified GCE (yellow line) in 0.5 M PBS (pH 3.0). Reprinted from [59].

Li et al. [59] modified GCE using thiolated polyaniline (tPANI), multi-walled carbon nanotubes (MWCNTs) and gold–platinum core–shell nanoparticles (Au@Pt). The bare GCE and prepared GCE-tPANI, GCE-tPANI-MWCNTs, GCE-tPANI-Au@Pt, and GCE-tPANI-Au@Pt-MWCNTs were tested as sensors to determine caffeine in the presence of other drug molecules including ascorbic acid, levodopa (LD), acetaminophen, diclofenac (DI), and acetylsalicylic acid. The results obtained by DPV highlighted GCE-tPANI-Au@Pt-MVCNT as the most suitable sensor for the detection of all six drug molecules (Figure 4). They explained this by the synergistic effect of all the used modifiers and their catalytic effects. The stability of the sensor was tested after 45 days of storage and the current peaks remained 95.3%, 95.2%, 104.1%, 97.79%, 95.70%, and 97.06% for AA, LD, AC, DI, AS, and CF, respectively. Thus, GCE-tPANI-Au@Pt-MVCNT was determined a stable sensor.

Pd nanoparticles, polymethyldopa (PMDA), and TiO_2_ nanoparticles were used as modifiers of the glassy carbon electrode TiO_2_@PMDA/Pd/GCE [60]. The capacity of the designed sensor for the simultaneous detection of CF and acetaminophen in phosphate buffer solution and in real samples was tested. The achieved value of the limit of detection is given in Table 1. The electropolymerization of poly(L-phenylalanine)-reduced graphene oxide on GCE led to the development of a new electrochemical sensor for the determination of CF and TP in an acidic environment (pH 1.7, 0.01M H_2_SO_4_) [61]. According to the DPV and CV measurements, P(L-Pal)/rGO/GCE proved to be a suitable sensor with good repeatability and reproducibility and high sensitivity towards the target analytes.

Horst et al. [62] used nanoparticles of bismuth and silver to prepare modified GCE (BiAgNPs/GCE) for the individual and simultaneous determination of caffeine, ascorbic acid, and paracetamol in phosphate buffer solution. The sensing performance of BiAgNPs/GCE was also validated in real pharmaceutical formulations for target analytes using differential pulse voltammetry. The obtained results of the LOD are presented in Table 1. 

Reddy et al. [63] presented the process of preparing a new sensor consisting of GCE whose surface was modified with Fe-doped MgNi_2_O_3_ nanoparticles. The aim of the research was to develop an electrochemical sensor capable of simultaneously detecting CF, dopamine, uric acid, and nicotine. Four separate peaks were observed using this sensor, which implied its good selectivity and sensitivity. The pH range from 3 to 10 was investigated and the results obtained by SWV showed that the optimum pH was 7 for the detection of target analytes. The authors noted that the interaction of CF with the Fe- MgNi_2_O_3_/GCE’s surface was the best at neutral and alkaline solutions because of the activity of OH^−^ ions with carbon C8 of the CF. In acidic solutions, it was assumed that the activity of H^+^ ions hindered the oxidation of CF. In the study by Varsha et al. [21], a glassy carbon electrode was modified with a nickel-based organic framework and reduced graphene oxide (Ni-MOF/RGO/GCE) for CF detection using the DPV method. The determination of caffeine was also observed at almost neutral pH conditions of the phosphate buffer solution. Both research groups observed a better CF response on the modified GCE compared to bare GCE, which was the result of the large specific surface area, surface porosity, and greater active sites on the modified electrodes [21,63]. In the case of Ni-MOF/RGO, the conjugated Π-electron system enabled its interaction with the aromatic ring of CF, leading to the adsorption of CF on the electrode’s surface. As a result, the oxidation peak was enhanced [21].

Murugan and Kumar [64] designed and investigated an electrochemical sensor for the simultaneous determination of paracetamol, tryptophan, and caffeine. The investigated sensor was designed via incorporating tin sulfide (SnS) and titanium dioxide (TiO_2_) on graphene oxide sheets (SnS/TiO_2_@GO ternary composite). This electrochemical sensor was utilized for the individual and simultaneous determination of paracetamol, tryptophan, and caffeine by using cyclic voltammetry and differential pulse voltammetry techniques. Electrochemical measurements with the SnS/TiO_2_@GO composite-modified glassy carbon electrode GC-SnS/TiO_2_@GO pointed to high activity toward the oxidation of paracetamol, tryptophan, and caffeine, whereas the large surface and high carrier mobility contributed to a significant decrease in overpotential. The increase in the concentration of paracetamol from 9.8 nM to 280 μM, of tryptophan from 13.3 nM to 157 μM, and of caffeine from 16.6 nM to 333 μM led to a linear increase in the current peak during the individual determination. 

Li and his coworkers [65] developed an efficient and eco-friendly ultraviolet (UV) irradiation method to synthesize ternary nanocomposites of gold-polyndole-reduced graphene oxide (Au-Pln-RGO), which were then used to fabricate an electrochemical caffeine (CF) sensor. The synthesis process, the polymerization of indole monomer, a reduction in Au^3+^ ions, and GO occurred simultaneously with the use of UV irradiation without using chemical compounds. The electrochemical behavior of caffeine was investigated using cyclic voltammetry and the results led to the conclusion that the Au-PIn-RGO nanocomposites effectively catalyzed the CF reaction on the electrode. The electrolyte solution and the scan rate had a significant effect on the oxidation peak of caffeine; therefore, a 0.1 M H_2_SO_4_ solution with a pH value of 0.7 was selected as the optimal solution. In order to evaluate the applicability of the Au-PIn-RGO/GCE sensor, the concentration of CF in a cola beverage sample was determined by the standard addition method and the recoveries were obtained in the range from 97.6 to 104.1%, which suggested that the proposed method can be used for the detection of caffeine in real samples.

A molecularly imprinted polymer membrane (MIP) based on cassava starch–Fe_3_O_4_ was investigated in order to detect acetaminophen and caffeine simultaneously [66]. The differential pulse voltammetry method was used during the investigation. This electrode was developed by the reaction of cassava starch with sodium tripolyphosphate as a crosslinking agent and acetaminophen and caffeine were added as templates. The sensitivity of the sensor was improved by the Fe_3_O_4_ nanoparticles. The highest sensitivity was achieved at a 2:2:1 ratio between cassava starch:STPP:acetaminophen/caffeine in the mixture for the MIP membranes. The voltammetry results showed that increasing the pH value led to the shift of the oxidation potential to a more negative direction. In addition, differential pulse voltammetry showed that the highest current peak occurs at pH 2. The sensors produced from the GCE modified with the MIP membrane from cassava starch–Fe_3_O_4_ can be used for the determination of the content of acetaminophen and caffeine in headache medicine with an accuracy of 96–99%. 

#### 2.1.2. Electrochemical Sensors Based on Carbon Paste Electrode

**Figure 5:**
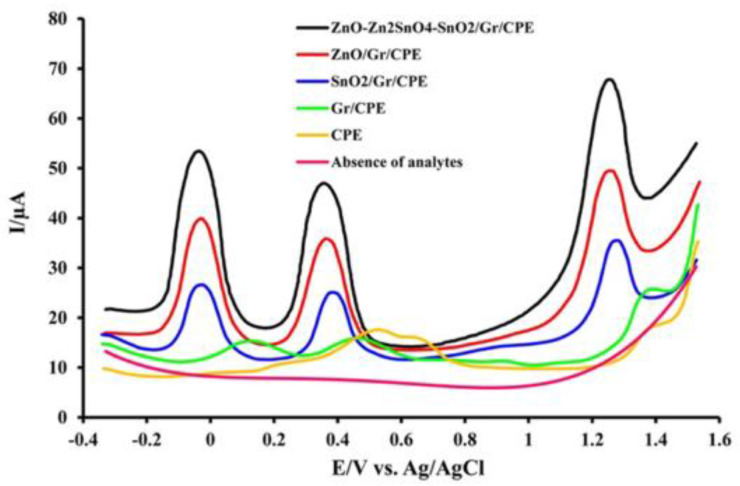
DPV of the bare CPE, Gr/CPE, SnO2/Gr/CPE, ZnO/Gr/CPE, and ZnO-Zn2SnO4-SnO2/Gr/CPE in the presence of 7.0 µM AA, 5.0 µM AC, and 6.0 µM CF, and ZnO-Zn2SnO4-SnO2/Gr/CPE in the absence of the analytes. Reprinted from [75].

In addition to GCE, carbon paste electrodes (CPE) and pencil graphite electrodes (PGE) have been frequently used for CF detection. Khoshhesab [74] modified a CPE electrochemical sensor with a nanocomposite of CuO and graphene (CuO-Gr/CPE) for the determination of CF, AC, and AA in a mixture. The nanocomposite modifier enhanced the sensor’s surface area and facilitated electron transfer in Britton–Robinson buffer solution. By analyzing the influence of the pH solution on the voltammetric response of CF and referring to the slope of the corresponding linear equation (E= −0.0532pH + 1.7155; R^2^ = 0.9772), it was concluded that an equal number of protons and electrons participated in the reaction. The achieved LOD for CF is shown in Table 2. The CuO-Gr/CPE with DPV proved to be adequate for the simultaneous determination of CF, AS, and AA with good repeatability (RSD 2.38%) and stability after 30 days of storage (retained CF peak 92.2%). Nikpanje et al. [75] demonstrated the preparation of modified CPE using graphene and a ZnO-Zn_2_SnO_4_-SnO_2_ nanocomposite. The capabilities of the electrochemical sensor for determining CF individually and simultaneously in the presence of ascorbic acid and acetaminophen in human urine and blood serum were investigated. The obtained LOD values (Table 2) for this sensor indicate its good properties and applicability for the detection of the target analyte in different matrices. Due to the synergistic effect of the nanocomposite and graphene as modifiers, the voltammetric response of CF on ZnO-Zn_2_SnO_4_-SnO_2_/Gr/CPE was improved to a greater degree than on the CPE as well as other CPE modifications, as illustrated in Figure 5.

Other researchers modified carbon paste electrodes with cerium oxide nanoparticles (CMCPE) [76] and cobalt oxide nanoparticles (Co(II, III) oxide) (NCOMCPE) [77] and tested them as sensors for the determination of CF in drugs and food samples. Different solutions, including PBS, H_3_PO_4_, HCl, and H_2_SO_4_, were investigated for their role as the supporting medium, and the best voltammetric response was obtained in a 0.01M sulfuric acid solution using NCOMCPE [77] and 0.1M PBS for CMPCE [76]. It is interesting that the researchers added a micellar solution to the basic solution and thus achieved a higher conductivity of the electrode because the micellar solution was adsorbed on the surface of the sensor. These results were confirmed by EIS measurements. It was suggested that the presence of SDS and nanoparticles affects the sensitivity of the electrode to CF and affected the kinetics of electron transfer. The highest oxidation peak of CF on the CV diagrams at pH 0.75 was due to the high conductivity of the electrodes and the achieved fast electron transfer [77]. In addition, cerium oxide nanoparticles improved the voltammetric response of CF in neutral PBS compared to the bare CPE [76]. By investigating the effect of the scan rate on the current peak of CF, Santhosh et al. [76] noticed a linear relationship between these parameters and indicated that the oxidation reaction of CF on CMCPE was an electrolytic redox process. On the other hand, Fekry et al. [77] observed a linear relationship between the current peak and the square root of the scan rate, thereby implying a diffusion-controlled process. In addition, the solution pH influenced the peak potential of CF. According to the Fekry et al. [77], a slope value of 28 mV/pH (dEp/dpH) suggested that different numbers of protons and electrons were included in the CF reaction on NCOMCPE. They hypothesized that some products of the oxidation process could block the surface of the sensor. By analyzing the data in the Table 2, it can be seen that good values of the LOD were achieved using the CMCPE and NCOMCPE sensors. However, future directions of CMCPE development should include the investigation of the application of this sensor in real samples, such as human body fluids, considering its biocompatibility, stability, and bactericidal activity.

Azab and his coworkers [78] investigated caffeine detection with a carbon paste electrode modified with a mixture of zeolite (Zeo) and multi-walled carbon nanotubes in a surfactant-improved medium with sodium dodecyl sulfate. Cyclic voltammetry, electrochemical impedance spectroscopy, and square wave voltammetry measurements were conducted as well as scanning electron microscopy to characterize the surface. Different beverage samples—tea, coffee, Nescafé, a carbonated beverage, and an energy drink—and a pharmaceutical sample in the form of Panadol Extra tablets were used. The appearance of the voltammetric response of caffeine on the bare CPE electrode at the potential of 1.46 V in 0.01 M H_3_PO_4_ solution was the consequence of the oxidation of the carbon atoms from the bare surface, but on the modified sensor, the potential shifted in a less positive direction (Table 2) and the voltammetric peak was intensified from 18 to 44 µA due to the synergistic effect of Zeo and MWCNTs. Nyquist plots confirmed the same results as those obtained through cyclic voltammetry. Among different media (H_2_SO_4_, H_3_PO_4_, HClO_4_, and a BR buffer) and at a scan rate of 50 mVs^−1^, the highest current peak was achieved in H_3_PO_4_ with a pH value of 1.0. For the simultaneous electrochemical determination of six redox-active biomolecules, namely, (AA), (DA), uric acid (UA), tryptophan (Trp), xanthine (XA), and CF, a nanocomposite that contained ferricyanide-doped chitosan and multi-walled carbon nanotubes (FC/Chi-MWCNTs) was synthesized [79]. This composite was incorporated with a graphite paste electrode (MGPE) for these measurements. The electrochemical method used for investigation was differential pulse voltammetry. The usage of MGPE in a suitable electrolyte solution provided six strong, stable, and continuous well-separated oxidation peaks towards electrooxidation at potentials of 0.15, 0.30, 0.45, 0.75, 0.85, and 1.34 V for AA, DA, UA, Trp, XA, and CF, respectively. 

#### 2.1.3. Electrochemical Sensors Based on Pencil Graphite Electrode

In the work of Bayraktepe et al. [80], the authors compared the electrochemical characteristics of an unmodified and modified pencil graphite electrode as a sensor for the simultaneous detection of four compounds: caffeine, chlorpheniramine maleate, ascorbic acid, and acetaminophen. Poly-L-methionine and gold nanoparticles were used to modify PGE. Scanning electron microscopy with energy-dispersive X-ray spectroscopy (SEM/EDX) and EIS showed an improved surface area of the modified PGE compared to the unmodified one. In addition, it was assumed that the electrocatalytic effect contributed to a better current response of the target analyte on AuNP-p(L-met)/PGE than on bare PGE.

The objectives of the work by Wong et al. [81] were to develop a PGE-based electrochemical sensor with palladium nanoparticles (PGE-PdNPs) and to investigate its ability to determine CF in different samples. The PGE-PdNPs had good analytical performance for the detection of CF in the presence of other compounds such as direct yellow 50, tryptophan, and carbendazim. Additionally, the modified PGE proved to be successful in the determination of the mentioned analytes both in synthetic urine and in river water (Table 3).

#### 2.1.4. Electrochemical Sensors Based on Boron-Doped Diamond Electrode

Sarakhman et al. [83] tested a miniaturized three-electrode system with an unmodified, thick-film, boron-doped electrode as a sensor electrode. This system proved to be efficient for both the individual and simultaneous rapid on-site determination of uric acid and caffeine. The initial measurements included the CV and LSV. The CV results enabled the determination of the most suitable pH value for the simultaneous determination of UA and CF, which provided pronounced peaks and a sufficiently large peak-to-peak difference; therefore, pH 3 was chosen as the most suitable. The effect of the scan rate was evaluated using LSV because of the irreversible oxidation processes and it was concluded that the electrochemical processes were diffusion-controlled. Square-wave voltammetry and differential pulse voltammetry were used for the detection of uric acid and caffeine and the parameters such as the LOD can be seen in the Table 4. Numerous interfering substances were included, such as K^+^, Na^+^, Mg^2+^, Ca^2+^, Cl^−^, SO_4_^2−^, ascorbic acid, dopamine, pyridoxine, glucose, and sucrose, and it was concluded that even though the tested organic compounds might affect the determination of UA and CF at high concentrations, the concentrations that were present in the real samples were much lower; thus, the tested system could be efficiently used. 

An anodically (AP-BDDE) and cathodically pretreated boron-doped diamond electrode (CP-BDDE) as an electrochemical sensor for the concomitant determination of caffeine, acetaminophen, and carisoprodol was discussed in the paper by Eisele et al. [84]. Since no difference was observed in the peak value of CF oxidation on AP-BDDE and CP-BDDE, the authors chose anodic pretreatment due to its simplicity and faster process execution over the cathodic pretreatment. It was concluded that AP-BDDE in combination with an appropriate voltammetric method had the ability to simultaneously quantify these three drug molecules available in pharmaceutical formulations without the pretreatment of the sample. The obtained LOD values and the electrochemical methods used are shown in Table 4.

#### 2.1.5. Electrochemical Sensors Based on Screen-Printed Carbon Electrode

Tyszczuk-Rotko and Szwagierek [85] prepared a bismuth screen-printed carbon electrode (BiF/SPCE) and Tyszczuk-Rotko et al. [86] used a commercially available screen-printed carbon electrode coated with carbon nanofibers (SPCE/CNFs) for the determination of caffeine. Differential pulse voltammetry [85] and differential pulse adsorptive-stripping voltammetry (DPAdSV) [86] were applied as suitable methods. Both tested sensors had good analytical performance for CF determination in 0.2 M H_2_SO_4_ [85] and 0.1 M H_2_SO_4_ [86] solutions as well as in real water and beverages samples. The LOD values for the developed sensors are presented in Table 5. The oxidation reaction of caffeine on SPCE/CNFs and on BiF/SPCE was irreversible. Tyszczuk-Rotko and Szwagierek [85] observed that the process was diffusion-controlled, while Tyszczuk-Rotko et al. [86] noted that the process was diffusion–adsorption-controlled. 

Serrano et al. [87] investigated and discussed the results obtained for the simultaneous determination of CF, paracetamol (PA), and ibuprofen (IB) using various sensors including screen-printed carbon electrodes (SPCE), multi-walled carbon nanotube-modified screen-printed electrodes (SPCNTE), carbon nanofiber-modified screen-printed electrodes (SPCNFE), and graphene-modified screen-printed electrodes (SPGPHE). Calibration plots for PA, IB, and CF were constructed in a 0.1 M acetate buffer with a pH value of 5.5, as this provided the best resolution and peak shape for the three selected analytes. The applicability of SPCNFE for the voltammetric analysis of real environmental samples was successfully demonstrated by the concomitant detection of PA, IB, and CF in spiked tap water samples, with the following recoveries: 103.1% for PA, 99.5% for IB, and 97.6% for CF. The limits of detection for caffeine using all four electrodes were calculated and are summarized in Table 5. According to the shown values, the highest sensitivity was obtained by using SPCNFE and SPGPHE.

Scanlon et al. [88] presented SPCE for the on-line detection of caffeine. These electrodes were based on graphite, were electrochemically treated using cyclic voltammetry, and were pre-anodized in various solutions to obtain an electrochemically active hydrophilic surface. The most suitable pretreatment in sulfuric acid was chosen for further electrode modification by Nafion. The Nafion-modified SPCE facilitated the improved detection of caffeine in acidic solutions and beverages.

Filik and Avan [89] demonstrated the possibility of using magnetic solid-phase microextraction (m-SPME) in combination with screen-printed carbon electrodes modified with poly-alizarin red S (ARS/SPCE) and square wave voltammetry to determine CF in real food and beverage samples. The goal was to provide the fast adsorption of the target analyte on the surface of the sensor as a simple pretreatment process. After that, the magnetic field allowed the magnetic sorbent to be separated from the solution and the adsorbed CF was eluted with the appropriate solution. The combination of electrochemical technique with the magnetic extraction of the solid phase as well as the use of a polymer as a sensor modifier proved to be very suitable for application in complex media. Various interferences including ions (Cl^−^, NO_3_^−^, SO_4_^2−^, PO_4_^3−^, Na^+^, K^+^, Ca^2+^, Fe^3+^, and Mg^2+^), amino acids (glycine, histidine, and L-tyrosine), and other substances such as ascorbic acid, vitamin B6, glucose, fructose, taurine, catechin, and epicatechin did not affect the selectivity of ARS/SPCE.

Lezi et al. [90] described the preparation process and the application of a graphene/Nafion-modified screen-printed electrode (SPE-Nafion/graphene) for CF determination in 0.2 M H_2_SO_4_/0.01 M HCl solution using adsorptive stripping voltammetry. Due to the synergistic effect of Nafion and graphene, the modified SPE exhibited high sensitivity and a low limit of detection for CF (Table 5). Additionally, SPE-Nafion/graphene was compared with GCE-Nafion/graphene and the authors concluded that SPE-Nafion/graphene had better performance regarding CF determination due to the higher surface area. Monteiro et al. [91,92,93] demonstrated the applicability of cork and graphene-based sensors for caffeine detection. Compared to other studies where researchers used several synthetic materials to prepare an electrochemical sensor, Monteiro et al. [91,92,93] used cork as a sustainable and renewable material. During the preparation of this electrochemical sensor, the mass ratio of cork and graphite was about 50 and 70%, and the cork–graphite electrode at a mass ratio of 70% showed a better sensibility for the determination of caffeine in aqueous solutions [91]. This electrode, with a mass ratio of 70% cork, was used to estimate the caffeine concentration in real samples (drugs and soft drinks). The results of the measurements clearly characterized this electrode as a novel tool for caffeine detection. The aim of the research undertaken by Boopathi et al. [94] was to develop a screen-printed electrochemical sensor based on a nanocomposite (molybdenum trioxide (MoO_3_) and graphitic carbon nitride sheets (GCNS)) for caffeine detection. The prepared sensor showed good stability after two weeks (current peak was 98.6% from its initial value), good reproducibility (RSD 2.9%), and good repeatability (RSD 3.2%).

Caffeine can often be found in medical pain relief formulations with acetaminophen and codeine. To avoid the side effects of the excessive use of such formulations, it is important to detect these compounds in biological fluids. Khairy et al. [95] and Mohamed et al. [96] developed stable, sensitive, and promising electrochemical sensors by modifying a screen-printed electrode with cerium-oxide nanoparticles (CeO_2_-SPE) and spongy graphene oxide (FSG-SPE), respectively. The authors tested modified SPEs for the detection of three analytes in Britton–Robinson buffer solution but under different pH values, namely, pH 2 [95] and pH 7 [96]. According to the electrochemical characterization of CeO_2_ NPs, Khairy et al. [95] determined the heterogeneous rate constant for both unmodified and modified SPE. The values of 1.80 × 10^−4^ and 3.20 × 10^−4^ cm s^−1^ for bare SPE and CeO_2_-SPE, respectively, suggested that the rate of charge transfer was easier on CeO_2_-SPE. This was also supported by the larger surface area of the modified electrode (6.73 × 10^−2^ cm^2^) compared to the unmodified one (5.12 × 10^−2^ cm^2^). In addition, Mohamed et al. [96] showed the improved responses of the analytes to modified SPE, which were attributed to the formed structure of the modifier. The FSG/SPE electrode in combination with the SWV method showed good analytical performance for detecting codeine, acetaminophen, and caffeine in their mixture in the supporting solution as well as blood plasma and the pharmaceutical formulation Solpadeine^®^ [96], while the applicability of CeO2-SPE in combination with DPV was confirmed in human serum [95]. 

#### 2.1.6. Electrochemical Sensors Containing Different Carbon Materials

The study conducted by Ferrag et al. [97] presented the process of preparing a carbon-ceramic electrode that was additionally modified with multi-walled carbon nanotubes and gold nanoparticles (MWCNT-AuNP-CCE). The developed sensor was examined for the determination of CF in the presence of xanthine and uric acid in phosphate buffer solution as well as in real samples. By comparing the Nyquist plots of CCE, CCE-MWCNT, CCE-MWCNT-Au, and CCE-MWCNT-AuNP, the authors concluded that CCE-MWCNT-AuNP had the lowest value of charge transfer resistance because it contained both MWCNT and AuNP modifiers. Therefore, the conductivity of CCE-MWCNT-AuNP was the highest, and this was the reason it achieved the best shape and highest intensity in the voltammetric responses of the analyzed compounds.

**Figure 6:**
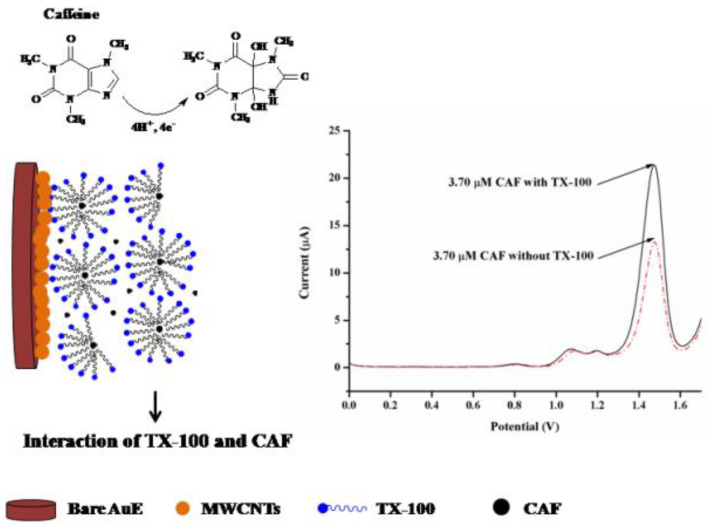
Illustration of interaction of CF with TX-100 ··MWCNTs/Au electrode and corresponding DPV of CF in in 0.1M acetate buffer (pH 3.0) at a scan rate of 50 mV/s without TX-100 and with 10 µL of 10 mM TX-100. Reprinted from [98].

Considering the good performance of multi-walled carbon nanotubes, e.g., in improving electrode sensitivity and facilitating electron transfer in electrochemical reactions, Mukdasai and Mukdasai [98] used them to modify Au electrodes. Besides MWCNTs, they also used Triton X-100 and prepared two electrochemical sensors for CF detection: MWCNTs/Au and TX-100-MWCNTs/Au. According to the differential pulse voltammogram (Figure 6), the current peak of CF was enhanced on TX-100-MWCNTs/Au due to the interaction of CF and C-H groups of TX-100. The pH of the solution affected the oxidation peak and optimal pH value, and the obtained value of the LOD is shown in Table 6. The selectivity of the DPV method was tested by detecting CF in the presence of ascorbic acid, NaCl, fructose, glucose, and xanthine, which did not interfere with the determination of caffeine.

Further, Monteiro et al. [92,93] used raw (RAC) and granulated cork (RGC) in the electrochemical sensor preparation process. The bare graphite (Gr) electrode is characterized by possessing the lowest electroactive sites when compared to GrRAC and GrRGC. Therefore, the current response of CF was lowest on bare GR. Due to the presence of cork as a modifier, the electroactivity of the sensor was enhanced. Phenolic, carboxyl, sulfonic, phosphate, and amino groups were the constituents of cork that enabled a positive electrode surface in an acidic environment (pH 4.4). Further, the interaction of CF and the protonated surface was potentiated by electrostatic interactions that contributed to the improved current response. The authors highlighted the superior capabilities of GrRAC compared to GrRGC because of the thermic treatment of GrRGC, which adversely affected its properties (phenolic and carboxylic groups were removed from its surface) [92]. In order to determine the effect of caffeine on the estradiol in child-bearing-aged women (18–35 yr), Raj and Goyal [99] developed a sensitive voltammteric biosensor that was based on the silver nanoparticles (AgNPs) and electrochemically reduced graphene oxide (ErGO) nanocomposite-modified pyrolytic graphite (AgNPs:ErGO/PG) [99]. The obtained results showed that the modified sensor exhibited an extraordinary electrocatalytic effect towards the oxidation of estradiol and caffeine, with a significantly pronounced current peak. The influence of ascorbic acid, xanthine, uric acid, and hypoxanthine as common metabolites was determined, and the results indicated that they did not interfere with the simultaneous determination of estradiol and caffeine. According to results of the study, AgNPs:ErGO/PG could be applied for the determination of the effect of caffeine on the concentration of estradiol in women of child-bearing age. It was observed that the increase in the estradiol level after the intake of ∼200 mg caffeine in blood serum and urine was in the range 10–42% and 11–38%, respectively. 

**Figure 7:**
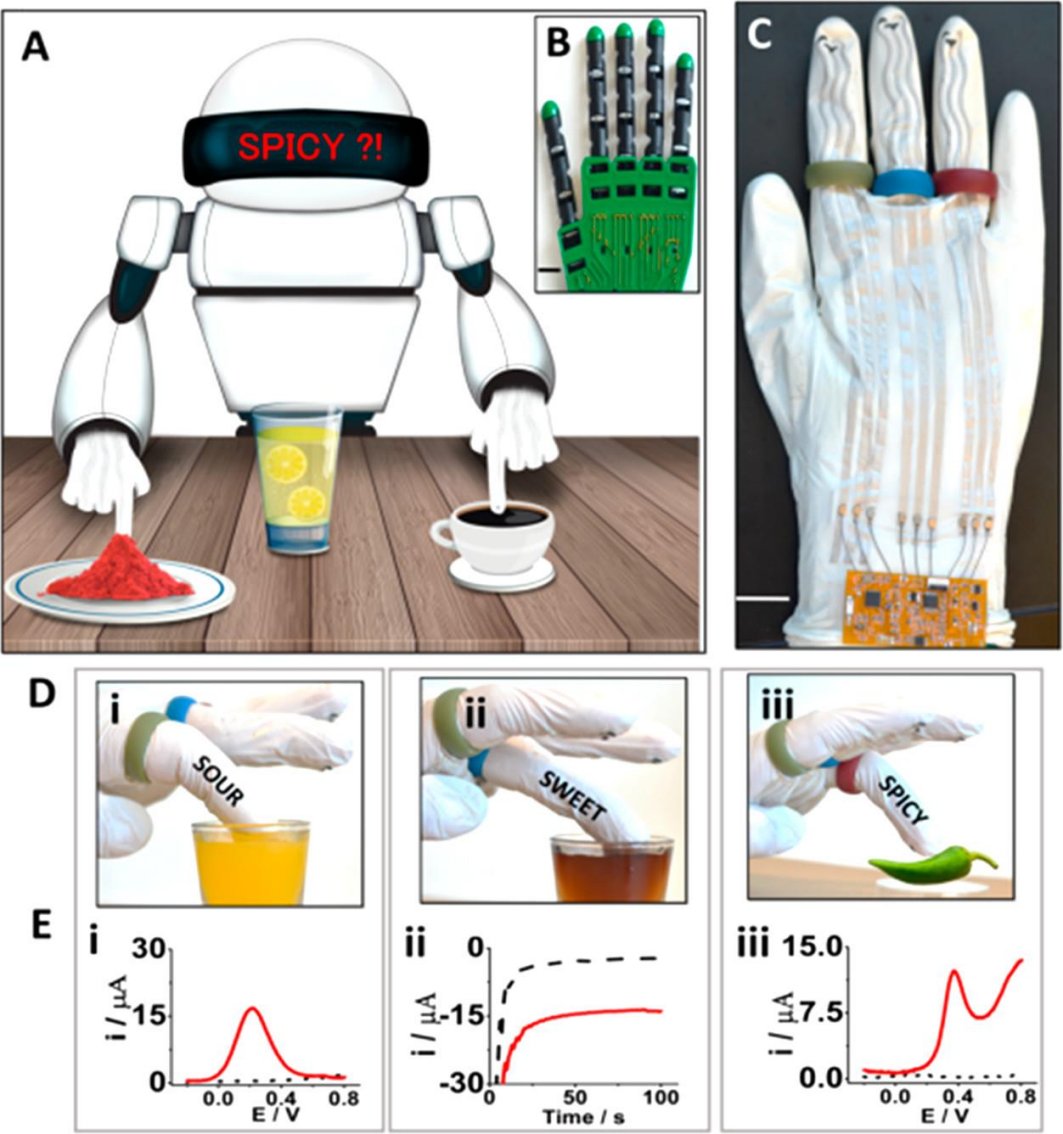
Robotic Sense fingers: schematics, fabrication, and analytical performance. (**A**) Schematics of robotic fingers immersed in lemon juice, coffee, and chili powder placed on the table for subsequent detection of sourness, caffeine, and spiciness, respectively. (**B**) Image illustrating the robotic hand used to develop the glove-based sense fingers. (**C**) Prototype of the screen-printed robotic sense fingers with long connections to the wrist where the electronic interface is located. Robotic fingers are identified with colored rings: green for carbon-printed sour-finger, blue for GOx Prussian blue carbon-printed sweet-finger, and red for carbon-printed spicy-finger. Scale bar, 5 cm. (**D**) Images and (**E**) corresponding electrochemical data of (**i**) robotic sour-finger dipped in orange juice and the SWV signature of ascorbic acid, (**ii**) robotic sweet-finger in cherry juice and amperometry data of glucose, and (**iii**) spicy-finger on green-pepper and SWV feedback response to the presence of capsaicin. Dotted lines correspond to the blank PBS response. Reprinted with permission from Ciui, B.; Martin, A.; Mishra, R.K.; Nakagawa, T.; Dawkins, T.J.; Lyu, M.; Cristea, C.; Sandulescu, R.; Wang, J. Chemical Sensing at the Robot Fingertips: Toward Automated Taste Discrimination in Food Samples. *ACS Sens.* **2018**, *3*, 2375–2384. https://doi.org/10.1021/acssensors.8b00778. Copyright {2022} American Chemical Society [100].

Ciui et al. [100] fabricated a novel hand-shaped robotic sensor for the observation of various substances such as ascorbic acid, glucose, capsaicin, and caffeine in complex matrices. A type of carbon-based ink was used to prepare the working electrode. An illustration of the developed robotic sensor (Figure 7) shows its ability to detect sourness, caffeine, and spiciness in the respective samples. Square wave voltammetry was carried out to determine CF. The appearance of a current peak at +1.2 V by SWV was consistent with other studies indicating the ability of this type of sensor to determine CF. Further, the prepared robotic sensor was tested for CF detection in coffee and decaffeinated coffee as well as in other beverage samples. According to the SWV results, the selectivity and reproducibility (RSD 8%) of the hand-shaped sensor for CF were very good. 

### 2.2. Mechanism of Caffeine Oxidation

**Scheme 1:**
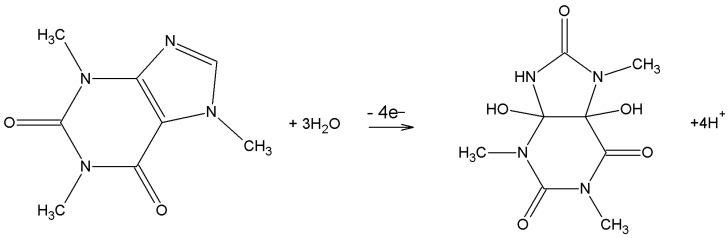
Mechanism of caffeine oxidation.

According to the available data from the literature, it is noted that the type and the pH value of the supporting solution have a remarkable effect on the current response of caffeine. The pH of the electrolyte is very important because it has a great effect on the shape of the voltammetric response and on the potential and current of the voltammetric response on the modified electrode, and it is very useful in the approximation of proton or electron ratios included in the electrode reaction. The Britton–Robinson, phosphate, and acetate buffer solutions as well as a sulfuric acid solution were most often used to determine caffeine individually and simultaneously in the presence of other molecules (Table 1, Table 2, Table 3, Table 4, Table 5, Table 6 and Table 7). In addition, the surfaces of the bare and modified electrochemical sensors have an effect on the mechanism of caffeine oxidation. According to Svorc [10], the same number of electrons and protons (4e^−^, 4H+) are involved in the process of caffeine oxidation (Scheme 1). The same conclusion was reached in the paper by Monteiro et al. [93], in which it was explained that the oxidation of the C-8 bond to the N-9 bond by 2H^+^, 2e^−^ and the formation of substituted uric acid occurs first. Further oxidation by 2H^+^, 2e^−^ led to the formation of the 4,5-diol analog of uric acid.

Jose et al. [57] explained that nitrogen atoms (N9) in the caffeine ring and the carbonyl oxygen atoms (O2 and O6) represent hydrogen acceptor sites. Sarakhman [83] discussed caffeine’s nature as a weak base (pKa~0.7), suggesting that nitrogen can be protonated in an acidic environment, while neutral species become dominant as the pH of the solution increases. These characteristics of caffeine caused the oxidation peak potential to remain unchanged as the pH increased from 4 to 5. Increasing the pH from 2 to 3 led to a shift in the peak potential in the positive direction, which implied the inclusion of protons in the oxidation process. Therefore, it can be assumed that the overall process of CF oxidation includes the loss of two electrons followed by the addition of an H^+^ and OH^−^ onto the double bond. In addition, it is assumed that a chemical reaction takes place in the solution at the same time, which leads to a loss of two more electrons and four protons. According to Geto and Brett [55], more acidic solutions were more convenient for the oxidation of caffeine than neutral or alkaline solutions. This conclusion was supported by the authors by the fact that the intensity of the CF current peak was the most pronounced at pH 2 (BR buffer solution). The peak potential of CF did not significantly change as the pH of the supporting solution increased, in contrast to other studies [83]. In addition, Anastasiadi et al. [53] concluded that in a BR buffer solution at a higher pH value of 8, a larger background current occurs due to the discharge of the electrolyte. Guan et al. [68] showed that the peak potential of CF remained unchanged with the increasing pH of PBS, indicating that protons were not included in the oxidation process.

In order to analyze the kinetics of the process, the researchers investigated the effect of the scan rate on the CF peak current. Depending on the type of sensor used, the researchers had different conclusions. Geto and Brett [55], Guan et al. [68], Jose et al. [57], and Monteiro et al. [93] noticed a linear relationship between the peak current (I_p_) and the square root of the scan rate (v^1/2^), indicating a diffusion-controlled process. On the other hand, Horst et al. [62] and Varsha et al. [21] observed a linear relationship between the peak current (I_p_) and the scan rate (v), suggesting that the process is adsorption-controlled. In the paper by Kumar et al. [58], the oxidation mechanism of caffeine on Co_3_O_4_ NS-modified GCE in an acetate buffer solution was described as a process controlled by diffusion and adsorption, while Santhosh et al. [76] indicated that it is only an electrolytic redox process and a quasi-reversible electron transfer between caffeine and the CMCPE electrode’s surface.

### 2.3. Other Electrochemical Sensors for Caffeine Determination

Borgul [101] tested an interesting solution that could replace carbon-based electrodes for detecting cocaine-cutting agents. They used the interface between two immiscible electrolyte solutions (ITIES)—in this case, a polarized liquid–liquid interface (LLI) between an aqueous sodium chloride solution of Britton–Robinson buffer and bis(triphenylphosphoranylidene)ammonium tetrakis(4-chlorophenyl)borate (BTPPATPBCl) solution in 1,2-dichloroethane — as a sensor and applied the cyclic voltammetry method. Caffeine is one of the most hydrophilic agents and should not pose an obstacle for cocaine detection using this method.

In addition, a gold-based sensor has been investigated for CF detection [102]. Synthetized gold nanoparticles (AuNPS) in a chitosan matrix were used as a modifier. Guan et al. [68] prepared a new electrochemical sensor based on covalent organic framework materials (COFs). COFs were chosen due to their large specific surface area, thermal stability, and pore modification capability. The compounds 1,3,6,8-tetra(4-formyl phenyl)pyrene (TFPPi) and 2,6-diaminopyridine (DP) were used for the synthesis of the COF. Gold nanoparticles (AuNPs) were also used in the preparation of this sensor and its ability was tested for simultaneously detecting theophylline and caffeine. The obtained limit of detection for CF is shown in Table 7.

**Figure 8:**
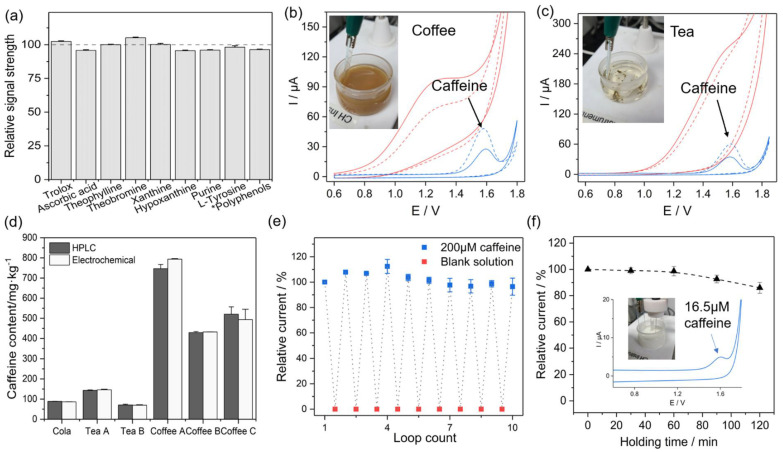
(**a**) Effects of related disruptors on the relative signal strength of caffeine (*: tea polyphenol concentration was calculated with catechin). (**b**,**c**) Cyclic voltammograms of the SNM/ITO (red) and *p*-PDMS/SNM/ITO (blue) in unprocessed coffee (**b**) and tea (**c**). To confirm the peak arising from caffeine, the CV curve was recorded before (solid line) and after (dashed line) caffeine’s addition. (**d**) Comparison of the Chinese national standard method and electrochemical method with respect to the detection of caffeine content in several commercially available foods. (**e**) Alternatively, CV scanning in 200 μM caffeine solution and blank solution. (**f**) Response of DPV current intensity to immersion time in milk with 16.5 μM caffeine. Inset: CV curve of *p*-PDMS/SNM/ITO in milk with 16.5 μM caffeine. Interference-free Detection of Caffeine in Complex Matrices Using a Nanochannel Electrode Modified with Binary Hydrophilic–Hydrophobic PDMS. Reprinted with permission from {Li, Y.; Luo, Z.; Li, G.; Belwal, T.; Li, L.; Xu, Y.; Su, B.; Lin, X. Interference-free Detection of Caffeine in Complex Matrices Using a Nanochannel Electrode Modified with Binary Hydrophilic−Hydrophobic PDMS. *ACS Sens.* **2021**, *6*, 1604–1612. https://doi.org/10.1021/acssensors.1c00004}. Copyright {2022} American Chemical Society [103].

Li et al. [103] demonstrated the process of the fabrication of a new electrochemical sensor using an indium tin oxide electrode with silica nanochannels on whose surface polydimethylsiloxane (PDMS) was deposited by plasma. A very important feature of PDMS was that it had both hydrophobic and hydrophilic oligomers. Therefore, the formation of hydrophilic–hydrophobic polymer structures hindered the transport of ions and molecules, except for amphipathic molecules such as caffeine. Furthermore, features such as anti-interference and anti-fouling allowed this sensor to be used in very complex media for CF detection without pretreatment (Figure 8). The voltammetric peak of CF continuously increased with the scan rate and the peak potential shifted to higher values. Based on the calculated parameters, the authors concluded that the CF oxidation process on the tested electrode could be described as a single electron transfer.

In addition, the work of Masibi et al. [104] demonstrated the preparation of a platinum electrode modified with bimetallic gold and silver nanoparticles and polypyrrole (PPY/AgAuBMNPs). The data obtained by electrochemical methods showed that the newly developed sensor was suitable for the detection of CF (Table 7). Arroyo-Gomez et al. [105] presented a different way of preparing electrochemical sensors for CF determination. They used the activated carbon from biomass as an electrode material that was activated with ZnCl_2_ and ZnCl_2_:FeCl_3_ (1:1). After the preparation of the two electrodes and the surface characterization, an electrochemical characterization was performed, which indicated a higher specific surface area of CA-ZnFe (0.018 cm^2^) compared to CA-Zn (0.005 cm^2^). Therefore, it was assumed that the voltammetric response of CF would be better on CA-ZnFe. By performing cyclic voltammetry, one irreversible anodic peak was recorded, which was in agreement with other studies. The DPV method was also incorporated into this research and according to the obtained voltammograms, a linear dependence between the CF concentration and the current peak was observed. The obtained values of the LOD for both electrodes are summarized in Table 7. The developed sensors were tested in real samples of CF containing beverages such as Coca-Cola and Pepsi. The recorded CF concentration values in these samples were very similar to the values obtained by the HPLC method, which confirms the applicability of these sensors.

By comparing the results obtained in the papers published by Ferrag et al. [97] and Masibi et al. [104], it can be said that the electrode modification process should be another key parameter for selecting a suitable sensor. Both fabricated sensors, MWCNT-AuNP-CCE and PPY/AgAuBMNPs, show applicability in real samples (Table 6 and Table 7) with low LOD values for CF, but Masibi and coworkers [104] chose a harmless modification process. In addition to obtaining good sensitivity, selectivity, and stability of the sensor, the authors should also work towards the use of environmentally friendly materials and substances in the modification processes.

In order to find an electrode for the selective detection of caffeine, moleculary imprinted polymers (MIPs) were synthesized [106]. The selectivity was tested by comparing the response of caffeine to the responses of theophylline, theobromine, and dopamine. The obtained sensors were used to measure the caffeine content in various samples using the Heat-Transfer Method (HTM). The HTM is a low-cost and simple thermal detection method based on thermal resistance differences at the solid–liquid interface. Moleculary imprinted polymers for the detection of caffeine were synthesized using the monomers of methacrylic acid (MAA), dopamine hydrochloride salt (99%), (hydroxyethyl) methacrylate (HEMA), and acrylamide (AA).

It is also possible to detect caffeine using biosensors such as Au/MPS/PAMAM G4.0-Au/CYP3A4, as shown in the paper published by Müller et al. [107]. They used CYP3A4 as a representative of cytochrome P450s, which are a large superfamily of heme-thiolate enzyme proteins that participate in caffeine metabolism. A gold electrode’s surface was modified by a 3-mercapto-propane-1-sulfonate (MPS) layer; then, poly-amido-amine (PAMAM) dendrimers were used as a connective layer between the gold electrode and the enzyme layer, with incorporated gold particles for enhanced conductivity and the enzyme layer as the top layer.

## 3. Conclusions—Prospects and Challenges

**Figure 9:**
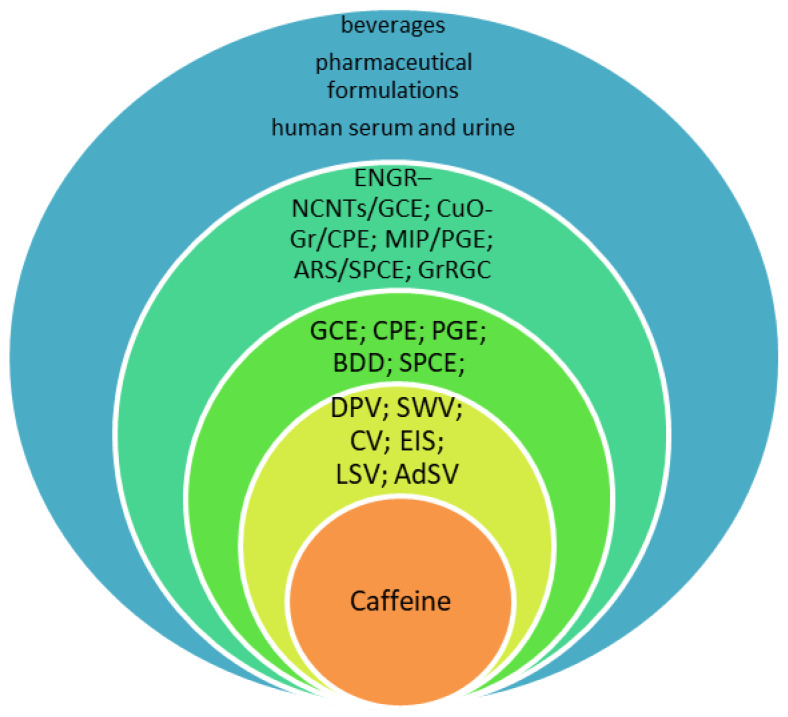
Illustration of the currently available data regarding methods, sensors, modified sensors, and tested real samples for caffeine determination.

Different electrochemical sensors with optimized voltammetric methods have been used for the determination of caffeine both in model solutions and in selected real samples. Based on the analyzed research, differential pulse voltammetry employing appropriate sensors was the method used most often. The researchers used various modifiers, including multi-walled carbon nanotubes, metal nanoparticles, and synthesized nanocomposites, to develop sensors that are suitable for the detection of caffeine in different mediums and for the achievement of low detection limits. An illustration of the currently available data regarding the methods, sensors, modified sensors, and analyzed real samples is presented in Figure 9. By analyzing the influence of the experimental parameters on the ability of the sensor, it can be concluded that depending on the electrode itself, other parameters should be optimized as well. The researchers noted that doping the carbon-based material with nitrogen and polymers, as well as incorporating the use of nanoparticles, led to improved electron transfer kinetics. However, one of the challenges faced by researchers when using metal nanoparticles as a modifier is the preparation of a stable sensor due to the possibility of their aggregation. Most of the research on caffeine quantification with electrochemical sensors has been carried out in an acidic environment. Moreover, most authors assumed that four protons and four electrons participate in the caffeine oxidation mechanism. Based on the presented literature data, the successful application of the developed sensors for the determination of caffeine can be observed. In the future, researchers should focus on methods and materials that are not harmful to the environment or even on the use of recycled materials. Thus, they would prevent the inadequate disposal of waste such as used batteries and contribute to the protection of the environment. In addition, the use of biomass and other sustainable and renewable materials for the production of electrochemical sensors would lead to a reduction in costs. Considering the constant advancement of technology, the challenge is to develop sensors that would be wearable and facilitate the quantification of caffeine, for which the latter will be automated. Recently, a robotic sensor with flexible fingers has been developed to analyze caffeine in food and beverage samples, and it would be desirable to continue research in this direction.

## Figures and Tables

**Table 1:** Electrochemical characteristics of glassy carbon electrode as sensor for caffeine determination.

Medium	Electrohemical Sensor	Method	LOD	CV Oxidation Peaks	Linear Range	Real Sample	References
0.1 M H_2_SO_4_	Pt@ZnCo_2_O_4_/GCE	CV, Amperometric i-t	0.0114 µM 0.01657 µM	+1.49 V	0.05–265.55 µM; 295.55–757.55 µM	Sugar-free cola beverage, energy drink water samples	[29]
BR buffer pH 1.8	Fe_2_O_3_/PEDOT/rGO/GCE	CV, DPV	0.33 μM	+1.35 V	1–800 μM	Energy beverages	[43]
0.1 M HClO_4_	rGO/Cu_2_O/Co(OH)2/GCE	CV, Amperometry	0.391 µM	+1.4 V	0.83–1200 µM	Caffeine-containing beverages and coffee powder 95.7–98.3% recovery	[44]
0.01 M H_2_SO_4_-Na_2_SO_4_ pH 1.70	PLCY/N-CNT/GCE	CV, CC, DPV	0.20 μM	/	0.40–140.0 μM	Tea, energy drink, pharmaceutical drug	[37]
0.1 M PBS pH 7	GO-CB-PEDOT:PSS/GCE	CV, SWV	3.4 μM	/	11–64 µM	Synthetic urine, and human serum samples	[42]
0.1 M PBS pH 2.5	GR/GCE	CV, SW AdSV	2.8 × 10^−7^ M * 3 × 10^−7^ M **	+1.39 V	2.6 × 10^−6^–2.1 × 10^−4^ M *; 2.6 × 10^−6^–1 × 10^−4^ M **	Food and beverage samples	[33]
0.1 M acetate buffer pH 7	ZnONPs/MGCE	CV, DPV	0.038 µM	+1.386 V	2–100 µM	Commercial beverage samples	[46]
0.1 M PBS pH 7	pPABSA/GCE	SWV	5.684 µM *; 11.951 µM **	+1.332 V (SWV)	10–100 mM	Commercial beverage samples	[67]
0.1 M PBS pH 4.5	P(L-Asp)/f-MWCNTs/GCE	SWV, CV	0.28 µM	+1.44 V	1–150 µM	Pharmaceutical formulations, blood serum, green tea	[47]
PBS pH 4.5	PFA/GR/GCE	CV DPV	0.08 µM	+1.44 V	1–160 µM	Tea and energy samples	[48]
0.1 M H_2_SO_4_ pH 1.5	Nafion/PDDA-MWCNT/GCE	CV DPV	0.05 µM	/	0.3–80 µM	Tea sample	[49]
0.1 M PBS pH 7	PDA/AuNPs/GCE	CV DPV	0.79 µM	+1.25 V	Upper limit 7.5 mM	Green tea samples	[51]
0.1 M H_2_SO_4_ pH 2	Anodically pretreated GC	DPV	5.80 µM	+1.46 V	1–200 μM	Human saliva	[53]
0.04 M BR pH 2	poly(7A4HN2SA)/GCE	DPV	0.23 µM	+1.34 V	10–500 μM	Coffee and beverages	[55]
0.1 M PBS pH 6.5	DP-Py COF/AuNPs/GCE	DPV	0.076 µM	+1.28 V (DPV)	30–700 µM	Black tea, paracetamol capsules	[68]
0.1 M PBS pH 7	GC/Gr/SiC-S@[Cu(pydc) (apym)]2	chronoamperometric	0.313 µM	/	20–100 µM	Commercial black tea and coffee samples	[56]
0.1 M KCl pH 2	NAHGO	DPV	0.008 µM	+1.5 V	0.01–40 µM	Energy drinks and pharmaceutical samples	[57]
0.1 M acetate buffer pH 4.4	Co_3_O_4_/GCE-Nafion electrode	SWV Chronoamperometric	0.23 µM 0.097 µM	+1.6 V	5–200 μM 4–500 μM	Tea samples	[58]
0.5 M PBS pH 3	GCE-tPANI-Au@Pt-MWCNT	DPV	5.0 µM	+1.37 V (DPV)	5–520 μM	Pharmaceuticals	[59]
0.2 M PBS pH 6	TiO_2_@PMDA/Pd/GCE	DPV	0.0239 µM	/	50 nM–4.5 μM	Human serum and pharmaceuticals	[60]
0.1 M PBS pH 7	GC-SnS/TiO2@GO	CV, DPV	4.4 nM	/	16.6 nM–333 μM	Human blood serum	[64]
0.1 M H_2_SO_4_ pH 0.7	Au-Pln-RGO/GCE	CV	0.26 μM	+1.56 V	0.8–1000 μM	Commercial beverages	[65]
PBS pH 2		CV, DPV	23 µM		50–900 µM	/	[66]
0.1 M PBS pH 7.2	GO/GCE	DPV	0.043 µM	+1.35 V	3.2–183 μM	Energy drinks	[69]
0.1 M PBS	MoS_2_/PANI@g-C_3_N_4_/GCE	DPV	0.062 µM	/	9.8–104 μM	Energy drink and pharmaceutical formulations	[70]
0.1 M PBS pH 7	3D NiO NWs/GCE	DPV	0.03 µM	+1.3 V	0.1–800 µM	/	[71]
0.1 M H_2_SO_4_ pH 1.7	P(L-Pal)/rGO/GCE	DPV	0.5 µM	+1.4 V	Both compounds 1–260 µM	Green tea, coffee and pharmaceutical tablets	[61]
0.1 M PBS pH 5	BiAgNPs/GCE	CV DPV	0.098 µM * 2.36 µM **	+1.32 V * +1.45 V **	2–40 µM	Pharmaceutical formulations	[62]
0.01 M H_2_SO_4_	Pt-GR/GCE	DPV	0.1129 µM	+1.336 V	0.09–0.38 mM	Tea samples	[72]
0.1 M PBS pH 7	Fe-MgNi_2_O_3_/GCE	CV SWV	0.276 µM	+1.354 V * +1.35 V ** (SWV)	50–4000 µM	Pharmaceuticalformulations, human urine, cigarettes, and coffee powder samples	[63]
0.1 M PBS pH 6.6	Ni-MOF/RGO/GCE	CV DPV	0.0409 µM 5.29 µM	+1.4 V	0.1–1 µM 10–250 µM	Coca-cola and pepsi	[21]
0.1 M PBS pH 3	MW-MOF-199/Naf-GCE	CV, DPV	1.2 µM	+1.25 V (DPV)	0.2 × 10^−6^–5 × 10^−6^ M	Commercial pharmaceutical tablets 95–108%	[20]
0.3 M HClO_4_ pH ∼1	poly(APTA)/SWNTs/GCE	amperometry	10 µM	+1.37 V	10–760 µM	Raspberry fruit tea recovery 97.54–99.12%	[40]
H_2_SO_4_/Na_2_SO_4_ pH 2.5	MnFe2O4@N-CNT	CV, SWV	0.83 × 10^−6^ M	+1.482 V	1.0 × 10^−6^–1.1 × 10^−3^ M	/	[35]
0.1 M PBS pH 7.4	P4VP–MWCNT/GCE	CV, DPV	1.19 nM *	+1.5 V	0.06–200 μM *	Pharmaceutical samples	[54]
0.1 M PBS pH 2.5	GR-NF/GCE	CV, SW-AdASV	3.8 × 10^−8^ M	+1.34 V * CV +1.44 V ** SW-AdASV	2.6 × 10^−7^–1.0 × 10^−5^ M	Pharmaceutical tablets	[52]
0.01 M H_2_SO_4_ pH 1.7	ENGR–NCNTs/GCE	CV, SWV	0.02 µM	/	0.06–50 µM	Pharmaceutical tablets	[34]
0.1 M PBS pH 7	Lt/fMWCNT/MGCE	CV, DPV	3.54 µM	+0.91 V	10.0–110.0 µM	Pharamceutical injection	[36]
0.1 M PBS pH 7	Nafion/ MWCNT-GCE	CV, DPV	0.04 µM	+1.32 V	/	Beverages and pharmaceutical samples	[50]
0.1 M H_2_SO_4_ pH 1.5	AT/Naf/GCE	CV, DPV	4.57 × 10^−8^ M	+1.41 V	0.1–4 µM	Pharmaceutical drug	[45]
0.1M PBS pH 7.4	GNP-CHIT-IL/r-GO/GCE	DPV, CV	4.42∙10^−9^ M	+1.05 V	2.50 × 10^−8^–2.49 × 10^−6^ M	Beverages and pharmaceuticals	[73]

*—individually; **—simultaneously.

**Table 2:** Electrochemical characteristics of carbon paste electrode as sensor for caffeine determination.

Medium	Electrohemical Sensor	Method	LOD	CV Oxidation Peaks	Linear Range	Real Sample	References
0.01 M H_3_PO_4_	ZMWCNTMCPE/SDS	CV, EIS, SWV, SEM	7.5∙10^−8^ M	+1.44 V	1.0 × 10^−5^– 5.0 × 10^−4^ M	Beverages and pharmaceutical tablets	[78]
0.04 M BR pH 7.2	ZnO-Zn_2_SnO_4_-SnO_2_/Gr/CPE	DPV chronoamperometry	0.00709 µM 0.00385 µM	/	0.02–97.51 µM	Urine and blood serum	[75]
0.01 M H_2_SO_4_ pH 0.75	NCOMCPE/SDS	CV DPV chronoamperometric	0.016 µM	/	5–600µM	Beverages and pharamceutical drugs	[77]
0.1 M PBS pH 7	CMCPE	CV DPV	0.036 µM	+1.26 V	5–80 µM	/	[76]
BR buffer pH 3.5	CuO-Gr/CPE	CV, DPV	0.010 mM	/	0.025–5.3 mmolL^−1^	Biological samples	[74]
0.1M PBS pH 4	FC/Chi-MWCNTs/MGPE	DPV	2.05 μM	+1.34 V	10–2439.02 µM	Human serum and urine	[79]

**Table 3:** Electrochemical characteristics of pencil graphite electrode as sensor for caffeine determination.

Medium	Electrohemical Sensor	Method	LOD	CV Oxidation Peaks	Linear Range	Real Sample	References
BR buffer pH 4	AuNP-p(L-met)/PGE	DPV	2.50 µM	/	8.3–2000 μM	Human serum	[80]
0.1 M H_2_SO_4_	PGE-PdNPs	SWV	3.9 µM	+1.402 V (SWV)	25–190 µM	River water and synthetic urine	[81]
PBS pH 4	MIP/PGE	CV, EIS. SWV	0.9 nM	/	2.0–1000.0 nmolL^−1^	Human fluids, pharamceuticals, and beverages	[82]

**Table 4:** Electrochemical characteristics of boron-doped diamond electrode as sensor for caffeine determination.

Medium	Electrohemical Sensor	Method	LOD	CV Oxidation Peaks	Linear Range	Real Sample	References
BR buffer pH 3	BDD	CV, LSV, SWV, DPV	4.6 µM (SWV) 2.1 µM (DPV)	+1.5 V	SWV 9.2–95.0 µM; DPV 4.6–95.7 µM	Human urine, human plasma	[83]
BR buffer pH 6	BDDE	CV, SWV, DPV	0.771 µM (SWV) 0.133 µM (DPV)	+1.50 V	2.99–84.8 µM	Pharmaceutical formulations	[84]

**Table 5:** Electrochemical characteristics of screen-printed electrodes as sensors for caffeine determination.

Medium	Electrohemical Sensor	Method	LOD	CV Oxidation Peaks	Linear Range	Real Sample	References
0.1 M H_2_SO_4_	SPCE/CNFs	CV, DP AdSV	5.6∙10^−8^ M	+1.25 V	2.0 × 10^−7^– 1.0 × 10^−6^ M	Commercial beverages	[86]
0.1 M acetate buffer pH 5.5	SPCE SPCNTE SPCNFE SPGPHE	DPV	1.4 µM 1.2 µM 0.4 µM 0.9 µM	/	4.8–93.3; 4.0–93.3; 1.2–6.4; 3.0–8.7 mgL^−1^	Real water samples	[87]
BR buffer pH 2	CeO_2_-SPE	CV, DPV	2.4 μM	+1.42 V	5–286 μM	Human serum	[95]
BR buffer pH 7	FSG-SPE	SWV	/	+1.50 V	1.10 × 10^−6^– 3.40 × 10^−4^ M	Blood plasma and pharmaceutical sample Solpadeine^®^	[96]
0.1 M acetate buffer solution pH 4	ARS/SPCE	CV, SWV	0.05 µM	+1.1 V(SWV)	0.5–20 µM	Food and beverage samples	[89]
0.20 M H_2_SO_4_/ 0.01 M HCl	SPE-Nafion/graphene	DP, AdSV, CV	0.021 µM	/	0.10–0.90 μM; 1.0–10 μM	Beverages	[90]
0.2 M H_2_SO_4_	BiF/SPCE	CV DPV	0.027 µM	+1.58 V	1 × 10^−7^– 2 × 10^−5^ M	Natural water samples	[85]
0.1 M PBS pH 7	MoO_3_@GCNS/SPCE	DPV	0.02124 µM	+1.407 V	0.5–359 μM; 410–810 μM	Green tea and coffee powder	[94]

**Table 6:** Electrochemical characteristics of other carbon-based electrodes as sensors for caffeine determination.

Medium	Electrohemical Sensor	Method	LOD	CV Oxidation Peaks	Linear Range	Real Sample	References
0.5 M H_2_SO_4_	GrRAC GrRGC	DPV	2.9 µM 6.1 µM	/	5–1000 μM	Soft drinks and pharmaceutical formulations	[93]
0.5 M H_2_SO_4_	GrRAC GrRGC	CV DPV	2.31 µM 6.33 µM	+1.54 V	0–50 μM	Pharmaceutical formulations and synthetic urine	[92]
0.5 M H_2_SO_4_	GrRAC-50% GrRAC-70%	CV, DPV	2.94 μM for GrRAC-70%	+1.51 V for both sensors	2.5–1000 μM	Pharmaceutical formulations and beverages	[91]
0.1 M PBS pH 6	MWCNT-AuNP-CCE	CV DPV	0.354 µM	+0.1 V (DPV)	10–1500 µM	Human serum and urine	[97]
0.1 M PBS pH 7.4	AgNPs:ErGO/PG	CV, SWV	0.54 nM	+1.275 V **	0.001–200 μM	Human urine and serum samples	[99]
0.1 M acetate buffer pH 3	TX-100-MWCNTs/Au	DPV	0.08 µM	+1.45 V (DPV)	0.58–11.50 µM	Tea samples	[98]

**—simultaneously.

**Table 7:** Electrochemical characteristics of other electrodes as for caffeine determination.

Medium	Electrohemical Sensor	Method	LOD	CV Oxidation Peaks	Linear Range	Real Sample	References
/	LLI	CV	1.57 × 10^−4^ M 1.32 × 10^−4^ M	/	100–500 µM	/	[101]
1 M H_2_SO_4_	CA-ZnFe CA-Zn	CV, DPV	1.60 × 10^−5^ M 2.85 × 10^−5^ M	+1.5 V both sensors	3.98 × 10^−5^– 6.37 × 10^−4^; 3.98 × 10^−5^– 4.58 × 10^−4^ M	Commercial beverages	[105]
0.4 M HClO_4_ pH 0.4	AuNP-CHIT modified gold electrode	CV DPV EIS	1.0 µM	+1.53 V	2.0 × 10^−6^–5.0 × 10^−2^M	Beverages	[102]
0.5 M K_2_SO_4_ and 0.1 M H_2_SO_4_	p-PDMS/SNM/ITO	DPV	0.02 µM	/	50 nM–700 μM	Coffee samples	[90]
0.0001 M PBS pH 3	PPY/AgAuBMNPs	SWV	2.02 µM	+1.38 V	0–59 µM	Energy drinks, coffee, tea	[104]

## Data Availability

Not applicable.
